# COVID-19 vaccine effectiveness against SARS-CoV-2 infection during the Delta period, a nationwide study adjusting for chance of exposure, the Netherlands, July to December 2021 

**DOI:** 10.2807/1560-7917.ES.2022.27.45.2200217

**Published:** 2022-11-10

**Authors:** Catharina E van Ewijk, Marjolein N Kooijman, Ewout Fanoy, Stijn FH Raven, Marit Middeldorp, Anita Shah, Brechje de Gier, Hester E de Melker, Susan JM Hahné, Mirjam J Knol

**Affiliations:** 1 Centre for Infectious Disease Control, National Institute for Public Health and Environment (RIVM), Bilthoven, the Netherlands; 2 European Programme for Intervention Epidemiology Training (EPIET), European Centre for Disease Prevention and Control (ECDC), Stockholm, Sweden; 3 Department of Infectious Diseases, Public Health Service Amsterdam-Amstelland, Amsterdam, the Netherlands; 4 Department of Infectious Diseases, Public Health Service Utrecht region, Utrecht, The Netherlands

**Keywords:** COVID-19, SARS-CoV-2, vaccine effectiveness, test-negative case-control, exposure, Netherlands

## Abstract

**Background:**

Differential SARS-CoV-2 exposure between vaccinated and unvaccinated individuals may confound vaccine effectiveness (VE) estimates.

**Aim:**

We conducted a test-negative case–control study to determine VE against SARS-CoV-2 infection and the presence of confounding by SARS-CoV-2 exposure.

**Methods:**

We included adults tested for SARS-CoV-2 at community facilities between 4 July and 8 December 2021 (circulation period of the Delta variant). The VE against SARS-CoV-2 infection after primary vaccination with an mRNA (Comirnaty or Spikevax) or vector-based vaccine (Vaxzevria or Janssen) was calculated using logistic regression adjusting for age, sex and calendar week (Model 1). We additionally adjusted for comorbidity and education level (Model 2) and SARS-CoV-2 exposure (number of close contacts, visiting busy locations, household size, face mask wearing, contact with SARS-CoV-2 case; Model 3). We stratified by age, vaccine type and time since vaccination.

**Results:**

VE against infection (Model 3) was 64% (95% CI: 50–73), only slightly lower than in Models 1 (68%; 95% CI: 58–76) and 2 (67%; 95% CI: 56–75). Estimates stratified by age group, vaccine and time since vaccination remained similar: mRNA VE (Model 3) among people ≥ 50 years decreased significantly (p = 0.01) from 81% (95% CI: 66–91) at < 120 days to 61% (95% CI: 22–80) at ≥ 120 days after vaccination. It decreased from 83% to 59% in Model 1 and from 81% to 56% in Model 2.

**Conclusion:**

SARS-CoV-2 exposure did not majorly confound the estimated COVID-19 VE against infection, suggesting that VE can be estimated accurately using routinely collected data without exposure information.

**Key public health message**

**What did you want to address in this study?**

People’s decisions to get vaccinated and whether or not to adhere to the public health COVID-19 control measures (people’s behaviour) might influence the measurement of vaccine effectiveness of the COVID-19 vaccinations. Persons who are vaccinated might also decide to have more close contacts with other persons because they consider themselves protected from COVID-19.

**What have we learnt from this study?**

People’s behaviour influenced the vaccine effectiveness estimates against COVID-19 only slightly.

**What are the implications of your findings for public health?**

To collect data on people’s behaviour is often time consuming and expensive. Our results suggest that we can relatively accurately estimate the COVID-19 vaccine effectiveness with routinely collected electronic health data without the need of collecting data on people’s behaviour.

## Introduction

Various vaccines to prevent coronavirus disease (COVID-19) were developed rapidly in response to the COVID-19 pandemic. In Europe, the European Medicines Agency granted market authorisation of the first COVID-19 vaccine (Comirnaty; BNT162b2 mRNA, BioNTech-Pfizer) in December 2020, followed by Vaxzevria (ChAdOx1-S, AstraZeneca) and Spikevax (mRNA-1273, Moderna) in January and COVID-19 Vaccine Janssen (Ad26.COV2-S, Janssen-Cilag International NV) in March 2021 [1-4]. The Netherlands started its COVID-19 vaccination campaign on 6 January 2021 [5]. 

Although efficacy of the mRNA (Comirnaty and Spikevax) and vector-based vaccines (Vaxzevria and Janssen) was proven in clinical trials, studies need to be conducted to monitor real-world effectiveness [6-9]. Many factors can confound or alter vaccine effectiveness estimates: intrinsic host factors such as age, sex and comorbidities, environmental factors such as geographical location and socioeconomic status which can manifest in differential risk of infection and vaccine uptake, and the chance of exposure such as the number of close contacts [10,11]. In addition, new variants of severe acute respiratory syndrome coronavirus 2 (SARS-CoV-2) have been shown to alter vaccine effectiveness [12]. Due to lack of randomisation, observational studies investigating vaccine effectiveness are prone to bias because of possible differences between vaccinated and unvaccinated individuals. Differences in the chance of exposure to SARS-CoV-2, such as the adherence to non-pharmaceutical interventions (NPI), might act as confounder of the vaccine effectiveness estimates. Persons who choose to adhere to NPI might be more likely to get vaccinated, leading to an overestimation of the vaccine effectiveness. On the other hand, vaccinated persons may feel they have a smaller risk of infection and therefore adhere less to NPI, leading to an underestimation of the vaccine effectiveness. 

COVID-19 vaccine effectiveness studies have been predominantly performed using routinely collected electronic health data. Although details are often available on age and sex, and sometimes on comorbidities and socioeconomic status, information on the individual’s exposure to SARS-CoV-2 is mostly lacking [13]. A close-contact cohort study that controlled for SARS-CoV-2 exposure showed lower vaccine effectiveness than studies based on routinely collected electronic health data, suggesting that differences in the chance of exposure to SARS-CoV-2 might have a substantial effect on vaccine effectiveness estimates [14].

We conducted a test-negative case–control study between 4 July and 8 December 2021, the period of circulation of the SARS-CoV-2 Delta variant (Phylogenetic Assignment of Named Global Outbreak (Pango) lineage designation B.1.617.2) to assess vaccine effectiveness of complete primary vaccination against SARS-CoV-2 in adults testing for SARS-CoV-2 at Public Health Service (PHS) testing facilities We also aimed to determine the effect of adjustment for potential confounders, in particular exposure to SARS-CoV-2, on the vaccine effectiveness estimates to asses if routinely collected data without exposure can be used instead.

## Methods

### Study design

We conducted a test-negative case–control study among adults attending SARS-CoV-2 PHS testing facilities throughout the Netherlands. Testing for SARS-CoV-2 at one of the PHS facilities was free of charge, has been widely available since 1 June 2020, and people could access them via car, bicycle or on foot throughout the Netherlands [15]. Appointments could be made by telephone or online. Testing was done either using a lateral-flow antigen test (LFAT), a reverse transcription PCR (RT-PCR) test or a loop-mediated isothermal amplification (LAMP) test. Testing was available for persons experiencing COVID-19 symptoms, for persons with known contact with a person who tested positive for SARS-CoV-2, or for travellers returning from countries that were classified by the Dutch government as high-risk areas for COVID-19 at the time. Persons who needed testing to travel or to access events were not allowed to test at PHS test facilities.

Individuals were invited to participate in the study via a link in the email confirmation the test appointment. Recruitment started on 8 February and ended on 11 March 2022. Individuals 18 years and older were eligible to participate if they completed the questionnaire before receiving test result, did not reside at a care facility and had not previously participated in the study. Participants who stated they had already received their test results were not able to fill in the questionnaire. 

### Data collection

The online questionnaire collected sociodemographic data (age, sex, education level [16], household size including the participant, country of birth and healthcare worker status) and information on clinical presentation (asymptomatic, respiratory and/or other non-respiratory symptoms), the presence of comorbidities, vaccination history, contact with a COVID-19 case in the 14 days before taking the survey, any previous (confirmed) COVID-19 episodes, and exposure-related variables (number of close contacts indoors and outdoors, whether or not they had been to a busy location indoors or outdoors 14 days before getting tested, and facemask wearing routine in public spaced indoors).

When making an appointment for testing at the PHS test facilities, people received a unique identifier in the appointment confirmation email. Participants were asked to fill in this unique identifier in the study questionnaire. Through this unique identifier, test results were later linked to the questionnaire data.

### Definitions

We defined a person as fully vaccinated if they received their second dose of Comirnaty, Spikevax or Vaxzevria vaccine ≥ 14 days before onset of symptoms (or before getting tested in case of asymptomatic infections or missing date of onset) or ≥ 28 days for those vaccinated with a single dose of Janssen vaccine [17]. Comorbidities were defined as diabetes type II, chronic lung disease, immunodeficiencies, heart disease, renal disease, liver disease, obesity (BMI > 30) or active cancer. Contact with a SARS-CoV-2-positive person was defined as one of the following types of contact with a SARS-CoV-2-positive person in the 14 days before getting tested: contact of > 15 min at > 1.5 m distance, contact of < 15 min at < 1.5 m distance or household contact. Close contacts (indoors and outdoors) were defined as any close contact with people at < 1.5 m distance for > 15 min, irrespective of the SARS-CoV-2 status of this person. Busy locations (indoors or outdoors) were defined as locations where the participant was not able to keep 1.5 m distance to others for > 15 min.

### Study population

This study focused on vaccine effectiveness of primary vaccination against the SARS-CoV-2 Delta variant. Therefore, we included participants who were tested between 4 July 2021 and 8 December 2021, the period in which the Delta variant caused virtually all infections [18]. During the study period, there was an Oxford stringency index for control measures between 32 of 100 (e.g. no restrictions on gatherings, no stay at home requirements, public events possible under certain conditions) on 4 July, which increased to 56 of 100 on 8 December (e.g. cancel public events, restrictions on gatherings, stay at home requirements) [19,20]. We included both symptomatic and asymptomatic persons. Only people who had an RT-PCR test were included. We excluded people who were partially vaccinated, received a booster (second vaccination in case of Janssen, third vaccination in case of the other vaccines) or third vaccination and those with heterologous vaccination schemes, with missing data regarding vaccination status, with onset of symptoms > 10 days before getting tested, people who reported a previous laboratory-confirmed SARS-CoV-2 infection, those with inconclusive test results and people who went to the test facility for confirmation of a positive self-administered LFAT to minimise recall bias.

### Statistical analysis

We compared characteristics between cases and controls and tested for significant differences using Pearson's chi-squared test. We compared the odds of vaccination between persons testing SARS-CoV-2-positive (cases) and -negative (controls). Vaccine effectiveness was calculated as 1 minus the odds ratio (OR). Firstly, OR and 95% confidence intervals (CI) were calculated using logistic regression, adjusting for age (spline), sex and calendar week (spline) (Model 1). Secondly, we additionally adjusted for comorbidity and education level (Model 2) to show the effect of these covariates separately from the exposure variables. Finally, we also adjusted for exposure through the following covariates as proxies: household size, number of close contacts indoors and outdoors, having visited busy locations indoors and outdoors, face mask wearing and contact with a SARS-CoV-2-positive person (Model 3). We selected the covariates a priori because of their presumed association with vaccination, infection or both.

We calculated vaccine effectiveness overall, per vaccine brand and per age group (18–49 and ≥ 50 years). In addition, we calculated vaccine effectiveness stratified by two age groups (18–49 and ≥ 50 years), vaccine type (mRNA and vector-based) and time since last vaccination (< 120 days and ≥ 120 days since completion of vaccination). We assessed whether differences in estimates between vaccine brands were significant (Model 3) and whether estimates of vector-based and mRNA-based vaccines were significantly different by time since vaccination (comparing < 120 days to ≥ 120 days in adults 18–49 and ≥ 50 years). Sensitivity analyses were performed restricting the data to symptomatic (at time of testing) individuals. Lastly, we estimated vaccine effectiveness including persons with previous SARS-CoV-2 infections in the analyses.

Statistical analyses were conducted using R version 4.0.2 (Vienna, Austria).

## Results

### Study population

From 4 July to 8 December 2021, 12,497 individuals participated, of whom 4,655 were excluded from analyses: persons tested with an LFAT or LAMP (n = 310), with previous laboratory-confirmed SARS-CoV-2 infection (n = 973), reporting a positive self-administered LFAT as reason for testing (n = 1,051), with symptom onset > 10 days before testing (n = 920), partially vaccinated (n = 1,083), heterologous vaccination (n = 35) or with three (or two in case of Janssen) vaccinations received (n = 75), and persons with missing data on vaccination (n = 208) (Figure).

**Figure:**
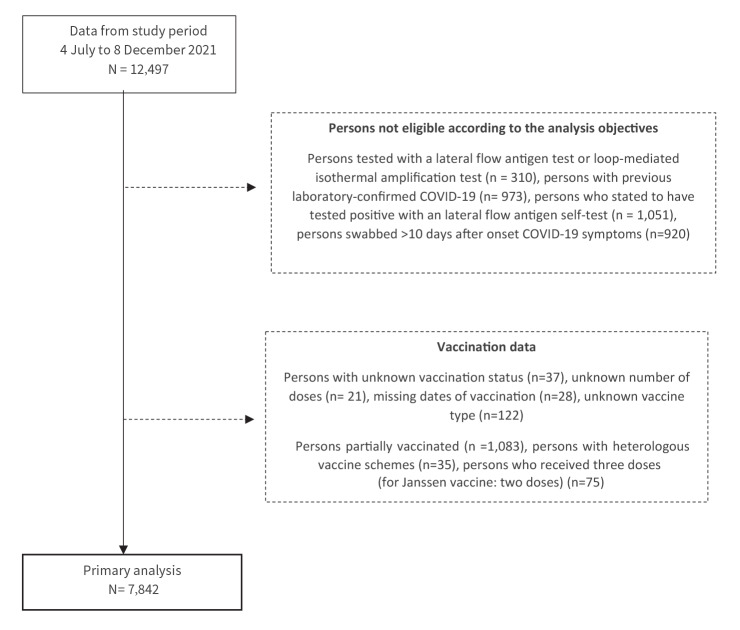
Flowchart of exclusions of participants in the COVID-19 vaccine effectiveness study, the Netherlands, 4 July–8 December 2021 (n = 12,497)

COVID-19: coronavirus disease.

A total of 7,842 participants were included of whom 7,127 (91%) were SARS-CoV-2-negative and 715 (9%) positive. Characteristics of the test-positive cases and test-negative controls are shown in Table 1.

**Table 1:** Characteristics and frequency of exposures of the study participants, COVID-19 vaccine effectiveness study, the Netherlands, 4 July–8 December 2021 (n = 7,842)

Variables	SARS-CoV-2 test results						p value^a^
	Negative			Positive			
	n	N	%	n	N	%	
Age group (years)							
18–29	1,210	7,127	17	114	715	16	0.014
30–44	1,521	7,127	21	129	715	18	
45–59	2,215	7,127	31	213	715	30	
60–69	1,522	7,127	21	171	715	24	
≥ 70	659	7,127	9	88	715	12	
Sex							
Male	2,285	7,104	32	280	714	39	< 0.001
Education level [16]							
Low	134	7,043	2	19	708	3	0.005
Middle	2,134	7,043	30	250	708	35	
High	4,775	7,043	68	439	708	62	
Country of birth							
The Netherlands	6,043	6,652	91	616	672	92	0.48
Other	609	6,652	9	56	672	8	
Comorbidities^b^							
Yes	1,171	7,127	16	120	715	17	0.81
Vaccination status							
Unvaccinated	292	7,127	4	83	715	12	< 0.001
Fully vaccinated	6,835	7,127	96	632	715	88	
Month of swab (2021)							
July	737	7,127	10	103	715	14	< 0.001
August	590	7,127	8	67	715	9.00	
September	762	7,127	11	33	715	5	
October	1,462	7,127	21	79	715	11	
November	3,105	7,127	44	372	715	52	
December	471	7,127	7	61	715	9	
Clinical presentation							
Asymptomatic	1,257	7,127	18	92	715	13	0.003
Respiratory (and other) symptoms	5,702	7,127	80	610	715	85	
Only other symptoms	168	7,127	2	13	715	2	
Healthcare worker							
Yes	1,115	7,127	16	124	715	17	0.24
Contact with SARS-CoV-2-positive person							
Yes	2,401	7,127	34	447	715	63	< 0.001
Household size (number of persons)^c^							
1	1,350	7,127	19	95	715	13	< 0.001
2–3	4,169	7,127	58	433	715	61	
≥ 4	1,608	7,127	23	187	715	26	
Facemask wearing							
Always	2,414	6,763	36	282	662	43	< 0.001
Mostly	1,414	6,763	21	162	662	24	
Sometimes	843	6,763	12	66	662	10	
Rarely	599	6,763	9	42	662	6	
Never	1,493	6,763	22	110	662	17	
Number of people with close contact indoors							
None	1,828	6,992	26	219	707	31	0.014
1–4 persons	1,916	6,992	27	204	707	29	
5–9 persons	1,325	6,992	19	118	707	17	
10–19 persons	899	6,992	13	71	707	10	
≥ 20 persons	1,024	6,992	15	95	707	13	
Number of people with close contact outdoors							
None	3,053	6,736	45	342	684	50	0.2
1–4 persons	1,832	6,736	27	170	684	25	
5–9 persons	822	6,736	12	73	684	11	
10–19 persons	437	6,736	7	45	684	7	
≥ 20 persons	592	6,736	9	54	684	8	
Visited busy locations indoors							
Yes	3,058	7,127	43	287	715	40	0.15
Visited busy locations outdoors							
Yes	1,064	7,127	15	125	715	17	0.07

COVID-19: coronavirus disease; SARS-CoV-2: severe acute respiratory syndrome coronavirus 2.

^a^ Pearson's chi-squared test.

^b^ Diabetes type II, chronic lung disease, immunodeficiencies, heart disease, renal disease, liver disease, obesity (BMI > 30) or cancer.

^c^ Including the participant.

Compared with controls, cases were more often male (39% vs 32% p < 0.001), older (36% ≥ 60 compared with 30% p = 0.014), had a lower education level (3% low and 35% middle vs 2% low and 30% middle p = 0.005), experienced more frequently respiratory symptoms (85% vs 80% p = 0.003) and were more often unvaccinated (11% vs 4%, p < 0.001). There were no significant differences in country of birth, presence of comorbidities, or the number of healthcare workers between controls and cases. Most cases were diagnosed in November, followed by July and October 2021. There was no change in testing protocol during the study period. 

### Vaccine effectiveness

In total 7,467 of 7,842 (95%) individuals were considered completely vaccinated: 4,817 (65%) with Comirnaty, 753 (10%) with Spikevax, 1,305 (17%) with Vaxzevria and 592 (8%) with Covid vaccine Janssen. The detailed characteristics and frequency of exposure by vaccine brand are provided in Supplementary Table 1.

Vaccine effectiveness against SARS-CoV-2 infection adjusting for age, sex, calendar week, education level, comorbidities and exposure (Model 3) was 64% (95% CI: 50–73). Adjusting for age, sex and calendar week only (Model 1) or in combination with education level and comorbidities (Model 2), but without the exposure variables, resulted in slightly higher estimates: 68% (95% CI: 58–76) and 67% (95% CI: 56–75), respectively (Table 2). The fully adjusted (Model 3) vaccine effectiveness for Comirnaty was 64% (95% CI: 51%–74), for Spikevax 76% (95% CI: 62–85), for Vaxzevria 53% (95% CI: 32–68) and for the Janssen vaccine 55% (95% CI: 32–70), showing slightly lower estimates than in Models 1 and 2. (Table 2). Adjusting for chance of exposure (Model 3) resulted in a decrease in vaccines effectiveness estimates compared with the model without exposure variables (Model 1) in adults aged 18–49 years: for example, the vaccine effectiveness of primary vaccination was 60% in adults 18–49 years when adjusted for chance of exposure (Model 3) and 66% without adjusting for exposure (Model 1), compared with 67% (Model 3) and 66% (Model 1) in adults ≥ 50 years (Table 2).

**Table 2:** Vaccine effectiveness of complete primary COVID-19 vaccination among adults against a positive SARS-CoV-2 test (Delta period), the Netherlands, 4 July–8 December 2021 (n = 7,842)

Vaccine status/brand	Number test-positive	Number test-negative	Vaccine effectiveness Model 1^a^	95% CI	Vaccine effectiveness Model 2^b^	95% CI	Vaccine effectiveness Model 3^c^	95% CI
All ages								
Unvaccinated	83	292	Reference		Reference		Reference	
Fully vaccinated	632	6,835	68%	58–76	67%	56–75	64%^d^	50–73
Comirnaty	387	4,430	70%	60–77	68%	57–76	64%^d^	51–74
Spikevax	40	713	79%	68–86	78%	66–86	76%^d^	62–85
Vaxzevria	145	1,160	58%	41–70	56%	39–69	53%^d^	37–68
Janssen	60	532	57%	38–71	55%	34–69	55%^d^	32–70
Age 18–49 years								
Unvaccinated	65	236	Reference		Reference		Reference	
Fully vaccinated	231	3,089	66%	53–76	65%	50–75	60%	43–72
Comirnaty	144	2,131	69%	57–83	68%	54–76	63%	45–75
Spikevax	30	450	72%	54–83	71%	53–83	68%	46–81
Vaxzevria	29	217	39%	0–63	36%	−5 to 61	30%	−21 to 60
Janssen	28	291	60%	35–76	59%	33–75	55%	23–74
Age ≥ 50 years								
Unvaccinated	18	56	Reference		Reference		Reference	
Fully vaccinated	401	3,746	66%	41–81	64%	35–80	67%	37–83
Comirnaty	243	2,299	67%	42–82	65%	37–81	68%	38–83
Spikevax	10	263	87%	69–94	85%	65–94	85%	63–94
Vaxzevria	116	943	61%	29–78	58%	23–78	62%	24–81
Janssen	32	241	51%	5–75	48%	−5 to 74	59%	11–81

CI: confidence interval; COVID-19: coronavirus disease; SARS-CoV-2: severe acute respiratory syndrome coronavirus 2.

^a^ Adjusted for age, sex and calendar week.

^b^ Adjusted for age, sex, calendar week, education level and comorbidities.

^c^ Adjusted for age, sex, calendar week, education level, comorbidities, household size, number of close contacts inside and outside, face mask wearing habits, visiting busy locations inside and outside, contact with a SARS-CoV-2-positive person.

^d^ Statistical significance between vaccine brands: Spikevax–Vaxzevria p < 0.01, Spikevax–Janssen p < 0.01, Spikevax–Comirnaty p < 0.05, Comirnaty–Vaxzevria p < 0.05, Comirnaty–Janssen p = 0.1, Vaxzevria–Janssen p = 0.8.

The mRNA vaccine effectiveness (Model 3) among people aged ≥ 50 years was 81% (95% CI: 66–91) at < 120 days and decreased significantly (p < 0.01) to 61% (95% CI: 22–80) at ≥ 120 days after vaccination. The vector-based vaccine effectiveness for this age group decreased (p = 0.1) from 69% (95% CI: 35–86) at < 120 days to 52% (95 CI: 4–76) at ≥ 120 days after vaccination. Among those aged 18–49 years, mRNA vaccine effectiveness decreased significantly (p < 0.05) from 70% (95% CI: 55–80%) at < 120 days to 57% (95% CI: 35–72%) at ≥ 120 days after vaccination. Vector-based vaccine effectiveness for this age group increased significantly (p < 0.05) from 17% (95% CI: −42 to 52%) at < 120 days to 65% (95% CI: 37–81%) at ≥ 120 after vaccination (Table 3). Estimates from Model 3 remained comparable to those from Models 1 and 2 without adjustment for chance of exposure.

**Table 3:** Vaccine effectiveness of complete primary COVID-19 vaccination among adults against a positive SARS-CoV-2 test (Delta period) the Netherlands, 4 July–8 December 2021 (n = 7,842)

Analysis type/vaccine type	Number test- positive	Number test negative	Vaccine effectiveness Model 1^a^	95% CI	Vaccine effectiveness Model 2^b^	95% CI	Vaccine effectiveness Model 3^c^	95% CI
Total population								
Unvaccinated	83	292	Reference		Reference		Reference	
mRNA < 120 days	128	2,578	79%	71–85	78%	69–84	74%	63–81
mRNA ≥ 120 days	299	2,565	60%	46–71	57%	41–69	56%	36–69
Vector-based < 120 days	77	613	52%	30–67	51%	29–66	51%	25–67
Vector-based ≥ 120 days	128	1,079	56%	37–69	53%	33–67	50%	27–66
Age 18–49 years								
Unvaccinated	65	236	Reference		Reference		Reference	
mRNA < 120 days	85	1,636	76%	65–83	75%	63–83	70%	55–80
mRNA ≥ 120 days	89	945	64%	45–76	62%	43–75	57%^d^	35–72
Vector-based < 120 days	34	178	29%	−15 to 56	28%	−18 to 56	17%	−42 to 52
Vector-based ≥ 120 days	23	330	69%	46–82	66%	42–81	65%^d^	37–81
Age ≥ 50 years								
Unvaccinated	18	56	Reference		Reference		Reference	
mRNA < 120 days	43	942	83%	67–91	81%	63–90	81%	61–91
mRNA ≥ 120 days	210	1,620	59%	26–77	56%	20–76	61%^d^	22–80
Vector-based < 120 days	43	435	62%	26–80	60%	22–80	69%	35–86
Vector-based ≥ 120 days	105	749	53%	13–74	49%	5–73	52%	4–76

CI: confidence interval; COVID-19: coronavirus disease; SARS-CoV-2: severe acute respiratory syndrome coronavirus 2.

^a^ Adjusted for age, sex and calendar week.

^b^ Adjusted for age, sex, calendar week, education level and comorbidities.

^c^ Adjusted for age, sex, calendar week, education level, comorbidities, household size, number of close contacts inside and outside, face mask wearing habits, visiting busy locations inside and outside, contact with a SARS-CoV-2-positive person.

^d^ Decreased or increased significantly (p < 0.05) compared with estimates at < 120 days after last vaccination.

In sensitivity analyses, estimates were similar if the analyses were restricted to persons reporting COVID-19 symptoms, except for the Janssen vaccine for which vaccine effectiveness estimates became 4–5 percentage points higher (Table 4). Estimates were slightly lower when we included people with previous SARS-CoV-2 infections in the analyses.

**Table 4:** Results from sensitivity analyses: Vaccine effectiveness of complete primary COVID-19 vaccination among adults against a positive SARS-CoV-2 test (Delta period), the Netherlands, 4 July–8 December 2021 (n = 8,319)

Analysis type/vaccine brand	Number test-positive	Number test-negative	Vaccine effectiveness Model 1^a^	95% CI	Vaccine effectiveness Model 2^b^	95% CI	Vaccine effectiveness Model 3^c^	95% CI
Symptomatic								
Unvaccinated	69	203	Reference		Reference		Reference	
Comirnaty	337	3,669	71%	60–79	69%	58–78	64%	49–75
Spikevax	38	589	79%	67–86	77%	64–86	75%	58–85
Vaxzevria	125	947	59%	40–72	57%	37–71	52%	26–69
Janssen	54	462	62%	43–75	59%	38–73	59%	35–74
Previous SARS-CoV-2 infections included								
Unvaccinated	88	363	Reference		Reference		Reference	
Comirnaty	394	4,650	66%	56–75	65%	53–73	59%	25–70
Spikevax	41	758	76%	65–84	76%	64–84	73%	59–83
Vaxzevria	146	1,231	54%	37–67	53%	34–66	48%	25–64
Janssen	62	586	54%	34–68	51%	29–66	50%	24–66

CI: confidence interval; COVID-19: coronavirus disease; SARS-CoV-2: severe acute respiratory syndrome coronavirus 2.

^a^ Adjusted for age, sex and calendar week.

^b^ Adjusted for age, sex, calendar week, education level and comorbidities.

^c^ Adjusted for age, sex, calendar week, education level, comorbidities, household size, number of close contacts inside and outside, face mask wearing habits, visiting busy locations inside and outside, contact with a SARS-CoV-2-positive person.

## Discussion

This study allowed to assess real-world vaccine effectiveness of COVID-19 vaccines adjusting for chance of SARS-CoV-2 exposure as potential confounder in addition to age, sex, calendar week, comorbidities and education level. The fully adjusted overall vaccine effectiveness against SARS-CoV-2 infection during Delta period was 64% (95% CI: 50–73). We used the number of close contacts inside and outside, face mask wearing habits, household size, whether busy locations inside and outside were visited, and contact with a SARS-CoV-2-positive person as proxy for chance of exposure to SARS-CoV-2. In our study, chance of exposure to SARS-CoV-2 did not majorly confound the estimation of COVID-19 vaccine effectiveness (only 2-6% difference).

The World Health Organization stated that it is essential to conduct real-world vaccine effectiveness studies taking into account various potential confounders, such as differences in risk-taking behaviour, in order to minimise bias due to differences between individuals who choose to get vaccinated and those who choose not to [11].

In our study population, unvaccinated individuals reported contact with a SARS-CoV-2-positive person more often than vaccinated individuals (50% vs 36%; p < 0.001). Differential exposure to a SARS-CoV-2-positive person between vaccinated and unvaccinated individuals is likely to confound vaccine effectiveness estimates, if not adjusted for. However, adjusting for these differences in the chance of SARS-CoV-2 exposure showed only a minor decrease in the vaccine effectiveness estimates. Potentially, individuals with more risk-taking behaviour (e.g. more close contacts) are at higher risk for other respiratory diseases besides SARS-CoV-2 and might more often take a test. Using a test-negative design might therefore inherently (partially) adjust for chance of exposure. Nonetheless, our findings in the Dutch setting suggest that vaccine effectiveness can be calculated relatively accurately using routinely collected electronic health data that lack information on individuals’ risk-taking behaviour, at least in a test-negative case–control study.

Vaccine effectiveness estimates of complete primary COVID-19 vaccination against infection (Model 1) were comparable to results of other test-negative case–control studies, which showed estimates between 69% and 88% for Comirnaty, between 73% and 82% for Spikevax, between 54% and 67% for Vaxzevria, and of 50% for the Janssen vaccine, and also higher estimates when looking only at symptomatic infections at time of testing [21-24]. Although estimates in these studies were adjusted for several covariates (age, sex, ethnicity, comorbidity and/or socioeconomic status), none were adjusted for determinants reflecting chance of exposure as potential confounder [21-24]. Household and close contact study designs allow standardisation of exposure by restricting analyses to households with at least one case or those with known contact with a COVID-19 case. The COVID-19 vaccine effectiveness results from such studies – associated with more intense exposure than within the community – generally show lower vaccine effectiveness than case–control or cohort studies that do not consider exposure [14,25,26]. This corresponds to the somewhat lower vaccine effectiveness in our Model 3, adjusting for chance of exposure. Adjusting for exposure decreased vaccine effectiveness in 18–49-year-old adults but not in people ≥ 50 years, possibly reflecting differences in risk-taking behaviour between these age groups: 18–49-year-olds reported contact with a SARS-CoV-2-positive person more often than ≥ 50 year-olds (38% vs 35% p < 0.05), more close contacts inside (17% vs 13% with 20 or more contacts, p < 0.001) and outside (11% vs 7% with 20 or more contacts, p < 0.001) and visited more often busy locations inside (48% vs 38%, p < 0.001) and outside (18% vs 13%p < 0.001). Not adjusting for chance of exposure might slightly overestimate the VE in this age group.

Vaccine effectiveness of mRNA vaccines decreased significantly over time with greater waning in adults aged ≥ 50 years. This is in accordance with results from a test-negative study in the United Kingdom that showed a greater decrease in vaccine effectiveness in adults 60 years and older (58.9% 2–9 weeks after vaccination to 36.6% at ≥ 20 weeks) compared with 40–64 year-olds (63.6% 2–9 weeks after vaccination to 57.8% at ≥ 20 weeks) [27].

For vector-based vaccines, however, we saw that vaccine effectiveness increased in adults 18–48 years ≥ 120 days after vaccination, compared with a decrease in adults ≥ 50 years. This difference in vaccine effectiveness over time between vector-based and mRNA vaccines has been demonstrated previously in the Dutch population [28,29]. Assuming that vaccine effectiveness against infection is predominantly dependent on antibody titres [30], the results could be explained by differences in titres over time, with antibodies initially rising faster after vaccination with mRNA vaccines compared with vector-based vaccines. That antibody levels rise more slowly has especially been seen for the Janssen vaccine [17]. This vaccine has predominantly been administered to the younger population in the Netherlands, which could explain the difference in vaccine effectiveness over time between age groups. In our study population, the share of Janssen vaccine among those who had received a vector-based primary vaccination was 57% in the age group 18–49 years vs 20% in the age group ≥ 50 years.

We also looked at bias from prior SARS-CoV-2 infection. Dutch research has shown good protection against infection with the SARS-CoV-2 Delta variant in individuals with either previous SARS-CoV-2 infection (76%) or primary vaccination (71%), but higher protection with both vaccination and previous infection (96%) [31]. Including individuals with immunity from previous COVID-19 in analyses may lead to underestimation of the vaccine effectiveness. For SARS-CoV-2, this gained immunity effect is greater in unvaccinated people [31]. In our study population, 17% of unvaccinated individuals reported a previous confirmed SARS-CoV-2 infection compared with 5% of the vaccinated (p < 0.001). Including these individuals in our analysis resulted in a slight decrease in the estimates, showing the importance of excluding, or adjusting for, previous infections. However, residual confounding might still be present if participants had (asymptomatic) infections without realising and/or testing for it.

We were able to determine the effect of adjustment for potential confounders, in particular chance of exposure to SARS-CoV-2, on the vaccine effectiveness estimates. Using a test-negative design allowed us to minimise ascertainment bias due to differences in healthcare seeking behaviour: individuals who are more likely to get vaccinated might also be more likely to get tested when experiencing COVID-19-like symptoms. In addition, inclusion of participants was prospective, and the participants needed to fill in the questionnaire before they knew their test results, thus reducing recall bias.

We used various behavioural characteristics such as the number of close contacts as proxies for chance of SARS-CoV-2 exposure. It is uncertain whether the factors are a good representation of exposure risk, and residual confounding might be present. Nonetheless, the number of close contacts has been used to predict the development of SARS-CoV-2 surges during the pandemic in the Netherlands and reflect transmission routes of SARS-CoV-2 and its control measures [32,33].

Furthermore, even though we included more than 7,800 participants in our study, this is only a small selection of the individuals who got tested at the PHS facilities during the study period. The study population is overrepresented by Dutch, highly educated, vaccinated and/or adults aged between 40 and 65 years and underrepresented by adults aged ≥ 65 years compared with the overall Dutch population [5,34,35]. This raises concerns about the generalisability of the results, since research showed that minorities and persons with a lower education level were likely to be more at risk for SARS-CoV-2 and less likely to be vaccinated [36,37]. Also, persons who are unwilling to get vaccinated might have different risk behaviour but might also be less inclined to get tested and/or participate in the study. This could lead to an overrepresentation of individuals who adhere to NPI and consequently an underestimation of the effect of chance of exposure as confounder.

We used self-reported vaccination status to calculate vaccine effectiveness, potentially leading to misclassification. As participants fill out the questionnaire before they know the test result, this misclassification will probably not depend on the outcome and may therefore lead to underestimation of the vaccine effectiveness.

Our results are based on data from the period when the SARS-CoV-2 Delta variant was dominant. It cannot be excluded that with a more transmissible variant such as Omicron, chance of exposure has more effect on the vaccine estimates than in our results. However, we believe that the principles of our methods remain the same regardless of the variant circulating and that the results are transferable to future variants.

## Conclusions

Overall, we found a moderate-to-high vaccine effectiveness against SARS-CoV-2 infections during the Delta period, with decreasing effectiveness by time since vaccination for the mRNA vaccines. Chance of exposure to SARS-CoV-2 confounded the estimation of COVID-19 vaccine effectiveness against SARS-CoV-2 infection only slightly, suggesting that COVID-19 vaccine effectiveness can be calculated relatively accurately using routinely collected electronic health data without exposure information.

## Supplementary Data

209000 Supplement

## References

[1] European Medicines Agency (EMA). EMA recommends first COVID-19 vaccine for authorisation in the EU. Amsterdam: EMA: 2020. Available from: https://www.ema.europa.eu/en/news/ema-recommends-first-covid-19-vaccine-authorisation-eu

[2] European Medicines Agency (EMA). EMA recommends COVID-19 vaccine Janssen for authorisation in the EU. Amsterdam: EMA: 2021. Available from: https://www.ema.europa.eu/en/news/ema-recommends-covid-19-vaccine-janssen-authorisation-eu

[3] European Medicines Agency (EMA). EMA recommends COVID-19 vaccine Moderna for authorisation in the EU. Amsterdam: EMA: 2021. Available from: https://www.ema.europa.eu/en/news/ema-recommends-covid-19-vaccine-moderna-authorisation-eu

[4] European Medicines Agency (EMA). EMA recommends COVID-19 vaccine AstraZeneca for authorisation in the EU. Amsterdam: EMA: 2021. Available from: https://www.ema.europa.eu/en/news/ema-recommends-covid-19-vaccine-astrazeneca-authorisation-eu

[5] National Institute for Public Health and the Environment (RIVM). Figures on the COVID-19 vaccination programme. Bilthoven: RIVM; updated 18 Jan 2022. Available from: https://www.rivm.nl/en/covid-19-vaccination/figures-vaccination-programme

[6] Baden LR El Sahly HM Essink B Kotloff K Frey S Novak R COVE Study Group . Efficacy and safety of the mRNA-1273 SARS-CoV-2 vaccine. N Engl J Med. 2021;384(5):403-16. 10.1056/NEJMoa2035389 33378609 PMC7787219

[7] Falsey AR Sobieszczyk ME Hirsch I Sproule S Robb ML Corey L Phase 3 safety and efficacy of AZD1222 (ChAdOx1 nCoV-19) Covid-19 Vaccine. N Engl J Med. 2021;385(25):2348-60. 10.1056/NEJMoa2105290 34587382 PMC8522798

[8] Polack FP Thomas SJ Kitchin N Absalon J Gurtman A Lockhart S Safety and efficacy of the BNT162b2 mRNA Covid-19 vaccine. N Engl J Med. 2020;383(27):2603-15. 10.1056/NEJMoa2034577 33301246 PMC7745181

[9] Sadoff J Gray G Vandebosch A Cárdenas V Shukarev G Grinsztejn B Safety and efficacy of single-dose Ad26.COV2.S vaccine against Covid-19. N Engl J Med. 2021;384(23):2187-201. 10.1056/NEJMoa2101544 33882225 PMC8220996

[10] Zimmermann P Curtis N . Factors that influence the immune response to vaccination. Clin Microbiol Rev. 2019;32(2):e00084-18. 10.1128/CMR.00084-18 30867162 PMC6431125

[11] World Health Organization (WHO). Evaluation of COVID-19 vaccine effectiveness: interim guidance, 17 March 2021. Geneva: WHO; 2021. Available from: https://apps.who.int/iris/handle/10665/340301

[12] Hahné S, Bollaerts K, Farrington P. Vaccination programmes epidemiology, monitoring, evaluation. 1 edition. London: Routledge; 2022. 470 p.

[13] Harder T Külper-Schiek W Reda S Treskova-Schwarzbach M Koch J Vygen-Bonnet S Effectiveness of COVID-19 vaccines against SARS-CoV-2 infection with the Delta (B.1.617.2) variant: second interim results of a living systematic review and meta-analysis, 1 January to 25 August 2021. Euro Surveill. 2021;26(41):2100920. 10.2807/1560-7917.ES.2021.26.41.2100920 34651577 PMC8518304

[14] Martínez-Baz I Trobajo-Sanmartín C Miqueleiz A Guevara M Fernández-Huerta M Burgui C Product-specific COVID-19 vaccine effectiveness against secondary infection in close contacts, Navarre, Spain, April to August 2021. Euro Surveill. 2021;26(39):2100894. 10.2807/1560-7917.ES.2021.26.39.2100894 34596016 PMC8485582

[15] Rijksoverheid. Juni 2020: Versoepeling coronamaatregelen en testen voor iedereen. [June 2020: Relaxation of corona measures and testing for everyone]. The Hague: Rijksoverheid; 2020. Dutch. Available from: https://www.rijksoverheid.nl/onderwerpen/coronavirus-tijdlijn/juni-2020-versoepeling-coronamaatregelen-en-testen-voor-iedereen

[16] Statistics Netherlands (CBS). Opleidingsniveau naar gemeenten, wijken en buurten. [Education level by municipality, district and neighbourhood]. The Hague: CBS. [Accessed: Feb 2022]. Dutch. Available from: https://www.cbs.nl/nl-nl/maatwerk/2020/17/opleidingsniveau-naar-gemeenten-wijken-en-buurten

[17] van den Hoogen LL Verheul MK Vos ERA van Hagen CCE van Boven M Wong D SARS-CoV-2 Spike S1-specific IgG kinetic profiles following mRNA or vector-based vaccination in the general Dutch population show distinct kinetics. Sci Rep. 2022;12(1):5935. 10.1038/s41598-022-10020-6 35396570 PMC8990276

[18] National Institute for Public Health and the Environment (RIVM). Variants of the coronavirus SARS-CoV-2. Bilthoven: RIVM; updated 14 Jan 2022. Available from: https://www.rivm.nl/en/coronavirus-covid-19/virus/variants

[19] Hale T Angrist N Goldszmidt R Kira B Petherick A Phillips T A global panel database of pandemic policies (Oxford COVID-19 Government Response Tracker). Nat Hum Behav. 2021;5(4):529-38. 10.1038/s41562-021-01079-8 33686204

[20] Relationship between number of COVID-19 cases and government response. Oxford: University of Oxford. [Accessed: 14 Jul 2022]. Available from: https://covidtracker.bsg.ox.ac.uk/stringency-scatter

[21] Lopez Bernal J Andrews N Gower C Gallagher E Simmons R Thelwall S Effectiveness of Covid-19 vaccines against the B.1.617.2 (Delta) variant. N Engl J Med. 2021;385(7):585-94. 10.1056/NEJMoa2108891 34289274 PMC8314739

[22] Sheikh A McMenamin J Taylor B Robertson C Public Health Scotland and the EAVE II Collaborators . SARS-CoV-2 Delta VOC in Scotland: demographics, risk of hospital admission, and vaccine effectiveness. Lancet. 2021;397(10293):2461-2. 10.1016/S0140-6736(21)01358-1 34139198 PMC8201647

[23] Tang P Hasan MR Chemaitelly H Yassine HM Benslimane FM Al Khatib HA BNT162b2 and mRNA-1273 COVID-19 vaccine effectiveness against the SARS-CoV-2 Delta variant in Qatar. Nat Med. 2021;27(12):2136-43. 10.1038/s41591-021-01583-4 34728831

[24] Kissling E Hooiveld M Martínez-Baz I Mazagatos C William N Vilcu A-M Effectiveness of complete primary vaccination against COVID-19 at primary care and community level during predominant Delta circulation in Europe: multicentre analysis, I-MOVE-COVID-19 and ECDC networks, July to August 2021. Euro Surveill. 2022;27(21):2101104. 10.2807/1560-7917.ES.2022.27.21.2101104 35620997 PMC9137272

[25] Lyngse FP Mølbak K Denwood M Christiansen LE Møller CH Rasmussen M Effect of vaccination on household transmission of SARS-CoV-2 Delta variant of concern. Nat Commun. 2022;13(1):3764. 10.1038/s41467-022-31494-y 35773247 PMC9244879

[26] de Gier B Andeweg S Backer JA Hahné SJ van den Hof S de Melker HE Vaccine effectiveness against SARS-CoV-2 transmission to household contacts during dominance of Delta variant (B.1.617.2), the Netherlands, August to September 2021. Euro Surveill. 2021;26(44):2100977. 10.2807/1560-7917.ES.2021.26.44.2100977 34738514 PMC8569927

[27] Andrews N Tessier E Stowe J Gower C Kirsebom F Simmons R Duration of protection against mild and severe disease by COVID-19 vaccines. N Engl J Med. 2022;386(4):340-50. 10.1056/NEJMoa2115481 35021002 PMC8781262

[28] National Institute for Public Health and the Environment (RIVM). Vaccine-induced protection against an infection with the Delta variant of the coronavirus. Bilthoven: RIVM; 2021. Available from: https://www.rivm.nl/en/news/vaccine-induced-protection-against-infection-with-delta-variant-of-coronavirus

[29] National Institute for Public Health and the Environment (RIVM) COVID-19 epidemiology and surveillance team. Effectiviteit van COVID-19-vaccinatie tegen SARS-CoV-2 infectie in de Delta periode. [Effectiveness of COVID-19 vaccination against SARS-CoV-2 infection in the Delta period]. Bilthoven: RIVM; 2021. Dutch. Available from: https://www.rivm.nl/documenten/effectiviteit-van-covid-19-vaccinatie-tegen-sars-cov-2-infectie-in-delta-periode

[30] Le Bert N Tan AT Kunasegaran K Tham CYL Hafezi M Chia A SARS-CoV-2-specific T cell immunity in cases of COVID-19 and SARS, and uninfected controls. Nature. 2020;584(7821):457-62. 10.1038/s41586-020-2550-z 32668444

[31] Andeweg SP de Gier B Eggink D van den Ende C van Maarseveen N Ali L Protection of COVID-19 vaccination and previous infection against Omicron BA.1 and Delta SARS-CoV-2 infections, the Netherlands, 22 November 2021- 19 January 2022. medRxiv. 2022:2022.02.06.22270457. 10.1101/2022.02.06.22270457 PMC9373894 35961956

[32] National Institute for Public Health and the Environment (RIVM). COVID-19 dataset. Bilthoven: RIVM. [Accessed: 17 Feb 2022]. Available from: https://data.rivm.nl/covid-19

[33] National Institute for Public Health and the Environment - Ministry of Health WaS. Modelling the spread of the coronavirus SARS-CoV-2 2021. Bilthoven: RIVM; updated 19 Nov 2021. Available from: https://www.rivm.nl/en/coronavirus-covid-19/modelling

[34] Statistics Netherlands (CBS). Herkomst. Hoeveel inwoners van Nederland zijn in het buitenland geboren? [Origin. How many residents of the Netherlands were born abroad?]. The Hague: CBS. [Accessed: 20 Oct 2022]. Dutch. Available from: https://www.cbs.nl/nl-nl/visualisaties/dashboard-bevolking/migratieachtergrond

[35] Statistics Netherlands (CBS) DCSO. Age distribution. The Hague: CBS. [Accessed: 17 Feb 2022]. Available from: https://www.cbs.nl/en-gb/visualisations/dashboard-population/age/age-distribution

[36] Coyer L Wynberg E Buster M Wijffels C Prins M Schreijer A Hospitalisation rates differed by city district and ethnicity during the first wave of COVID-19 in Amsterdam, The Netherlands. BMC Public Health. 2021;21(1):1721. 10.1186/s12889-021-11782-w 34551752 PMC8456400

[37] Coyer L Boyd A Schinkel J Agyemang C Galenkamp H Koopman ADM Differences in SARS-CoV-2 infections during the first and second wave of SARS-CoV-2 between six ethnic groups in Amsterdam, the Netherlands: A population-based longitudinal serological study. Lancet Reg Health Eur. 2022;13:100284. 10.1016/j.lanepe.2021.100284 34927120 PMC8668416

