# Supplementary material for: COVID-19 vaccine effectiveness against SARS-CoV-2 infection during the Delta period, a nationwide study adjusting for chance of exposure, the Netherlands, July to December 2021
Source: Euro Surveill. 2022 Nov 10;27(45):2200217. doi: 10.2807/1560-7917.ES.2022.27.45.2200217 (PMC9650707; doi:10.2807/1560-7917.ES.2022.27.45.2200217)
Supplement: Supplement [file 22-00217_EWIJK_supplement.pdf]

This supplementary material is hosted by *Eurosurveillance* as supporting information alongside the article "COVID-19 vaccine effectiveness against SARS-CoV-2 infection during the Delta period, a nationwide study adjusting for chance of exposure, the Netherlands, July to December 2021" on behalf of the authors, who remain responsible for the accuracy and appropriateness of the content. The same standards for ethics, copyright, attributions and permissions as for the article apply. Supplements are not edited by *Eurosurveillance* and the journal is not responsible for the maintenance of any links or email addresses provided therein.

Supplementary Table 1 - **Characteristics and frequency of exposures of the study participants by vaccine brand (N=7567), The Netherlands, 4 July to 8 December 2021.**

	Vaccine brand								
Variables	Comirnaty		Spikevax		Vaxzevria		Janssen		p value[1]
	N = 4,817		N = 753		N= 1,305		N = 592		
	n / N	%	n / N	%	n / N	%	n / N	%	
Test result									<0.001
Negative	4,430 / 4,817	92%	713 / 753	95%	1,160 /1,305	89%	532 / 592	90%	
Positive	387 / 4,817	8.0%	40 / 753	5%	145 / 1,305	11%	60 / 592	10%	
Age group (years)									<0.001
18-29	775 / 4,817	16%	137 / 753	18%	96 / 1,305	7%	161 / 592	27%	
30-44	1,107 / 4,817	23%	232 / 753	31%	108 / 1,305	8%	90 / 592	15%	
45-59	1,456 / 4,817	30%	334 / 753	44%	223 / 1,305	17%	335 / 592	57%	
60-69	761 / 4,817	16%	36 / 753	5%	871 / 1,305	67%	5 / 592	1%	
70+	718 / 4,817	15%	14 / 753	2%	7 / 1,305	1%	1 / 592	0.2%	
Sex									<0.001
Male	1,639 / 4,801	34%	200 / 748	27%	432 / 1,305	33%	180 / 592	30%	
Education level									<0.001
Low	106 / 4,762	2%	7 / 743	1%	24 / 1,295	2%	7 / 585	1%	
Middle	1,446 / 4,762	30%	169 / 743	23%	436 / 1,295	34%	160 / 585	27%	
High	3,210 / 4,762	67%	567 / 743	76%	835 / 1,295	64%	418 / 585	71%	
Country of birth									0.11
Dutch	4,085 / 4,505	91%	638 / 709	90%	1,120 / 1,211	92%	510 / 552	92%	
Other	420 / 4,505	9%	71 / 709	10%	91 / 1,211	8%	42 / 552	8%	
Comorbidities [a]									<0.001
Yes	813 / 4,817	17%	109 / 753	14%	303 / 1,305	23%	40 / 592	7%	
Month of swab (2021)									<0.001
July	424 / 4,817	9%	70 / 753	9%	121 / 1,305	9%	107 / 592	18%	
August	371 / 4,817	8%	61 / 753	8%	126 / 1,305	10%	50 / 592	8%	
September	505 / 4,817	10%	80 / 753	11%	117 / 1,305	9%	65 / 592	11%	
October	993 / 4,817	21%	142 / 753	19%	249 / 1,305	19%	111 / 592	19%	
November	2,176 / 4,817	45%	347 / 753	46%	610 / 1,305	47%	227 / 592	38%	

Variables	Vaccine brand								p value[1]
	Comirnaty		Spikevax		Vaxzevria		Janssen		
	N = 4,817		N = 753		N= 1,305		N = 592		
	n / N	%	n / N	%	n / N	%	n / N	%	
December	348 / 4,817	7%	53 / 753	7%	82 / 1,305	6%	32 / 592	5%	
Clinical presentation									0.07
Asymptomatic	811 / 4,817	17%	126 / 753	17%	233 / 1,305	18%	76 / 592	13%	
Respiratory (and other) symptoms	3,895 / 4,817	81%	613 / 753	81%	1,037 / 1,305	79%	507 / 592	86%	
Only other symptoms	111 / 4,817	2%	14 / 753	2%	35 / 1,305	3%	9 / 592	2%	
Health care worker									<0.001
Yes	566 / 4,817	12%	157 / 753	21%	347 / 1,305	27%	106 / 592	18%	
Contact with SARS-CoV-2 positive person									0.73
Yes	1,697 / 4,817	35%	280 / 753	37%	471 / 1,305	36%	213 / 592	36%	
Household size (no. of persons)									<0.001
1	918 / 4,817	19%	121 / 753	16%	248 / 1,305	19%	92 / 592	16%	
2-3	2,834 / 4,817	59%	399 / 753	53%	902 / 1,305	69%	300 / 592	51%	
4+	1,065 / 4,817	22%	233 / 753	31%	155 / 1,305	12%	200 / 592	34%	
Facemask wearing									0.04
Always	1,696 / 4,567	37%	254 / 720	35%	468 / 1,229	38%	177 / 561	32%	
Mostly	963 / 4,567	21%	169 / 720	23%	255 / 1,229	21%	112 / 561	20%	
Sometimes	564 / 4,567	12%	99 / 720	14%	138 / 1,229	11%	66 / 561	12%	
Rarely	389 / 4,567	9%	58 / 720	8%	100 / 1,229	8%	63 / 561	11%	
Never	955 / 4,567	21%	140 / 720	19%	268 / 1,229	22%	143 / 561	25%	
No. of people with close contact indoors									0.1
None	1,235 / 4,728	26%	175 / 741	24%	367 / 1,283	29%	154 / 576	27%	
1-4 persons	1,345 / 4,728	28%	202 / 741	27%	326 / 1,283	25%	148 / 576	26%	
5-9 persons	875 / 4,728	19%	138 / 741	19%	254 / 1,283	20%	114 / 576	20%	
10-19 persons	582 / 4,728	12%	118 / 741	16%	161 / 1,283	13%	68 / 576	12%	
20 or more persons	691 / 4,728	15%	108 / 741	15%	175 / 1,283	14%	92 / 576	16%	
No. of people with close contact outdoors									<0.001
None	2,098 / 4,564	46%	311 / 712	44%	596 / 1,229	48%	230 / 560	41%	
1-4 persons	1,197 / 4,564	26%	205 / 712	29%	345 / 1,229	28%	148 / 560	26%	
5-9 persons	544 / 4,564	12%	92 / 712	13%	147 / 1,229	12%	81 / 560	14%	
10-19 persons	293 / 4,564	6%	47 / 712	7%	78 / 1,229	6%	48 / 560	9%	
20 or more persons	432 / 4,564	10%	57 / 712	8%	63 / 1,229	5%	53 / 560	10%	
Visited busy locations indoors									0.01

	Vaccine brand								
Variables	Comirnaty		Spikevax		Vaxzevria		Janssen		p value[1]
	N = 4,817		N = 753		N= 1,305		N = 592		
	n / N	%	n / N	%	n / N	%	n / N	%	
Yes	2,104 / 4,817	44%	341 / 753	45%	513 / 1,305	39%	270 / 592	46%	
Visited busy locations outdoors									<0.001
Yes	702 / 4,817	15%	111 / 753	15%	173 / 1,305	13%	124 / 592	21%	

[1] Pearson's Chi-squared test; Fisher's exact test
